# The effects of a histone deacetylase inhibitor on biological behavior of diffuse large B-cell lymphoma cell lines and insights into the underlying mechanisms

**DOI:** 10.1186/1475-2867-13-57

**Published:** 2013-06-07

**Authors:** Ying Cai, Wenli Cui, Weixiang Chen, Ping Wei, Yayun Chi, Ping Zhang, Rui Bi, Xiaoyan Zhou

**Affiliations:** 1 Department of Pathology, Fudan University Shanghai Cancer Center, Shanghai, PR China; 2 Department of Oncology, Shanghai Medical College, Fudan University, Shanghai, PR China; 3 Institute of Pathology, Fudan University, Shanghai, PR China; 4 Cancer Research Institute, Fudan University Shanghai Cancer Center, Shanghai, PR China

**Keywords:** Diffuse large B-cell lymphoma, HDAC, Trichostatin A, Akt pathway, p53

## Abstract

**Background:**

Epigenetic control using histone deacetylase (HDAC) inhibitors is a promising therapy for lymphomas. Insights into the anti-proliferative effects of HDAC inhibitors on diffuse large B-cell lymphoma (DLBCL) and further understanding of the underlying mechanisms, which remain unclear to date, are of great importance.

**Methods:**

Three DLBCL cell lines (DoHH2, LY1 and LY8) were used to define the potential epigenetic targets for Trichostatin A (TSA)-mediated anti-proliferative effects via CCK-8 assay. Cell cycle distribution and apoptosis were detected by flow cytometry. We further investigated the underlying molecular mechanisms by examining expression levels of relevant proteins using western blot analysis.

**Results:**

TSA treatment inhibited the growth of all three DLBCL cell lines and enhanced cell cycle arrest and apoptosis. Molecular analysis revealed upregulated acetylation of histone H3, α-tubulin and p53, and dephosphorylation of pAkt with altered expression of its main downstream effectors (p21, p27, cyclin D1 and Bcl-2). HDAC profiling revealed that all three cell lines had varying HDAC1–6 expression levels, with the highest expression of all six isoforms, in DoHH2 cells, which displayed the highest sensitivity to TSA.

**Conclusion:**

Our results demonstrated that the HDAC inhibitor TSA inhibited DLBCL cell growth, and that cell lines with higher expression of HDACs tended to be more sensitive to TSA. Our data also suggested that inhibition of pAkt and activation of p53 pathway are the main molecular events involved in inhibitory effects of TSA.

## Background

Diffuse large B-cell lymphoma (DLBCL) is the most common type of non-Hodgkin’s lymphoma. Rituximab, an anti-CD20 antibody, administered as induction or maintenance therapy in combination with CHOP significantly prolonged event-free survival of DLBCL. However, continued use of rituximab has resulted in CD20 negative transformation of tumor cells and failure to demonstrate benefit [1]. Therapeutic challenges persist, and investigations of new targeted strategies are urgently needed.

The histone deacetylase (HDAC) enzymes remove acetyl groups from histone and non-histone proteins, and lead to the formation of a compacted and transcriptionally-repressed chromatin structure. As a result, the global gene expression profile is modified and cellular function is altered via multiple pathways. Aberrant HDAC expression in cancers suggests that HDACs are potential targets for epigenetic treatment [2].

Class 1 and 2 histone deacetylase expression in a panel of lymphoma cell lines and tissue sections was previously reported [3], and clinical evaluation indicates that lymphoid malignancies are more sensitive to HDAC inhibitors compared to other solid tumors [4]. Accordingly, HDAC inhibitors have been widely used in clinical trials in lymphoma, including peripheral T-cell lymphoma, mantle cell lymphoma, and DLBCL [5]. Furthermore, HDAC inhibitors, e.g. Romidepsin (Istodax, Celgene, Summit, USA) and Vorinostat (Zolinza, Merck, Whitehouse Station, USA), have been accepted by the US FDA for treating advanced and refractory cutaneous T-cell lymphoma (CTCL) [6].

Although clinical trials have proven suppressing effects of selected inhibitors on DLBCL patients, no HDAC inhibitors have been approved for the treatment of DLBCL [7]. Insights into the anti-proliferative effects of HDAC inhibitors on DLBCL, and further understanding of the underlying mechanisms are of great importance. In this study, we evaluated the effects of Trichostatin A (TSA), a hydroxamic acid derivative that inhibits most HDAC isoforms, and elucidated the molecular mechanisms underlying the subsequent altered biological behavior of DLBCL cell lines. We identified varied expression levels of HDACs in DoHH2, LY1 and LY8 cell lines, and thus we selected these lines for our investigation.

## Results

### Effects of TSA on growth inhibition in all three DLBCL cell lines induced by cell cycle arrest and apoptosis

**Figure 1:**
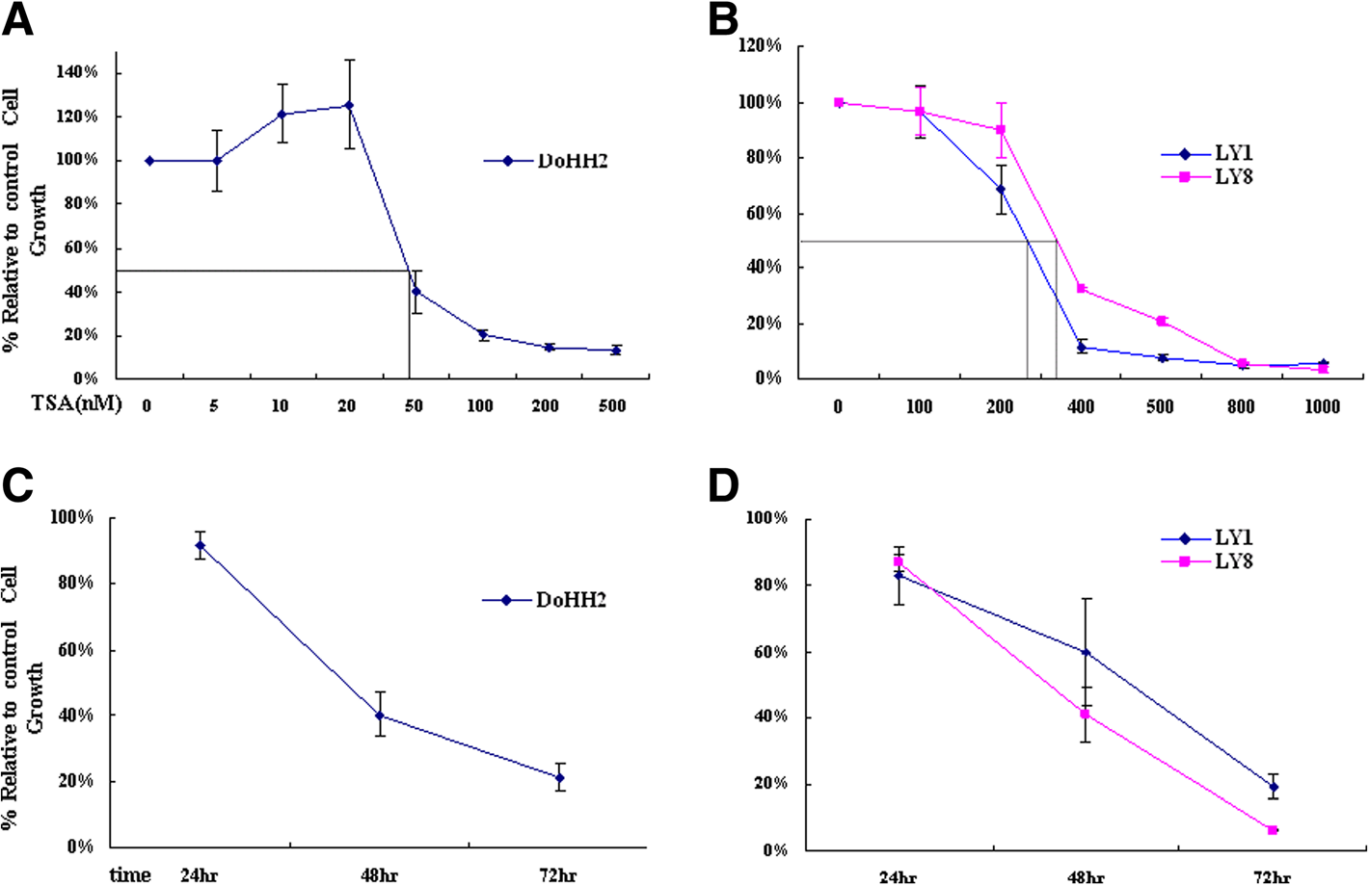
**Growth inhibition of DLBCL cell lines by TSA. **(**A**) DoHH2, (**B**) LY1 and LY8 cells were treated with the indicated concentrations of TSA for 48 h to examine dose-dependent inhibitory effects. Probit Regression determined IC_50 _values of 45 nM, 250 nM and 350 nM in DoHH2, LY1 and LY8 cells, respectively. (**C**) Cell growth curves of DoHH2 (treated with 50 nM TSA), (**D**) LY1 and LY8 cells (treated with 300 nM TSA). Cells treated with DMSO were used as controls. Cell viability of DoHH2, LY1 and LY8 cells was determined by CCK-8 assay. Results are displayed as mean ± SD of three independent experiments performed in triplicate.

Three DLBCL cell lines (DoHH2, LY1 and LY8) were treated with varying concentrations of TSA. Growth of all three DLBCL cell lines was inhibited by TSA treatment in a dose-dependent manner (Figure 1A, B). A much higher drug concentration was needed to significantly inhibit the growth of both LY1 and LY8 cells compared with DoHH2 cells. Probit Regression analysis of results after 48 h of TSA treatment revealed IC_50_ values for LY1, LY8 and DoHH2 cells of 250 nM, 350 nM and 45 nM, respectively, indicating DoHH2 cells as the most sensitive to TSA. From these results, we selected a treatment level for DoHH2 cells of 50 nM TSA, and 300 nM TSA for LY1 and LY8 cells for all subsequent experiments. After 48 h treatment, the relative cell viability of DoHH2, LY1 and LY8 cells declined to 40%, 60% and 41%, respectively, and declined further to 21%, 19% and 6% after 72 h treatment, indicating that TSA exhibits its inhibitory effects in DLBCL cells in a time-dependent manner (Figure 1C, D).

**Figure 2:**
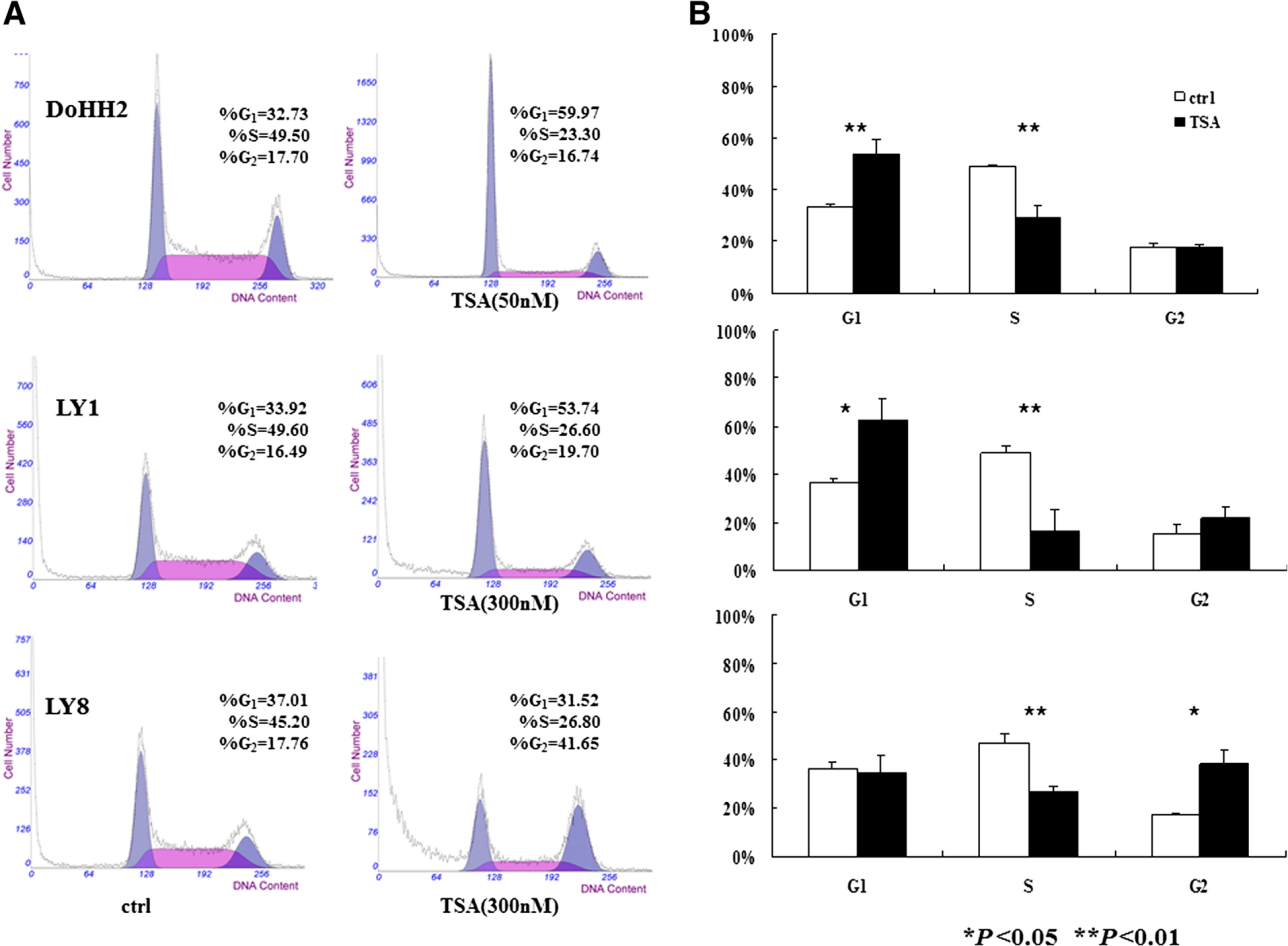
**Effects of TSA on cell cycle in DLBCL cells. **(**A**) PI staining and flow cytometric analysis of cell cycle distribution in DoHH2, LY1 and LY8 cells after treatment with vehicle or indicated concentration of TSA after 24 h. One representative experiment is shown for each cell line. (**B**) Results displayed as mean ± SD of three independent experiments performed in triplicate. * *P* < 0.05 and ** *P* < 0.01 compared with vehicle treatment.

We next examined the cell cycle phase distribution after TSA treatment. The percentage of untreated DoHH2 cells at G_1_ phase was 32.73%, which increased to 59.97% after 24 h TSA treatment, while the percentage of S phase cells decreased from 49.50% to 23.30%. The percentage of LY1 cells in G_1_ phase increased from 33.92% to 53.74% after TSA treatment, while S phase cells declined from 49.60% to 26.60% after 24 h treatment. However, in LY8 cells, the percentage of G_2_ phase cells increased from 17.76% to 41.65%, and S phase decreased from 45.20% to 26.80%, indicating a G_2_/M arrest (Figure 2A). A significant G_0_/G_1_ arrest was induced in DoHH2 cells after 24 h treatment relative to control cells, with a corresponding decrease of cells in S phase (*P* < 0.01) (Figure 2B). A consistent induction of G_0_/G_1_ arrest (*P* < 0.05) and corresponding S phase reduction (*P* < 0.01) were observed in LY1 cells after 24 h treatment (Figure 2B). However, we detected a G_2_/M arrest (*P* < 0.05) and relevant S phase decline (*P* <0.01) in LY8 cells (Figure 2B).

**Figure 3:**
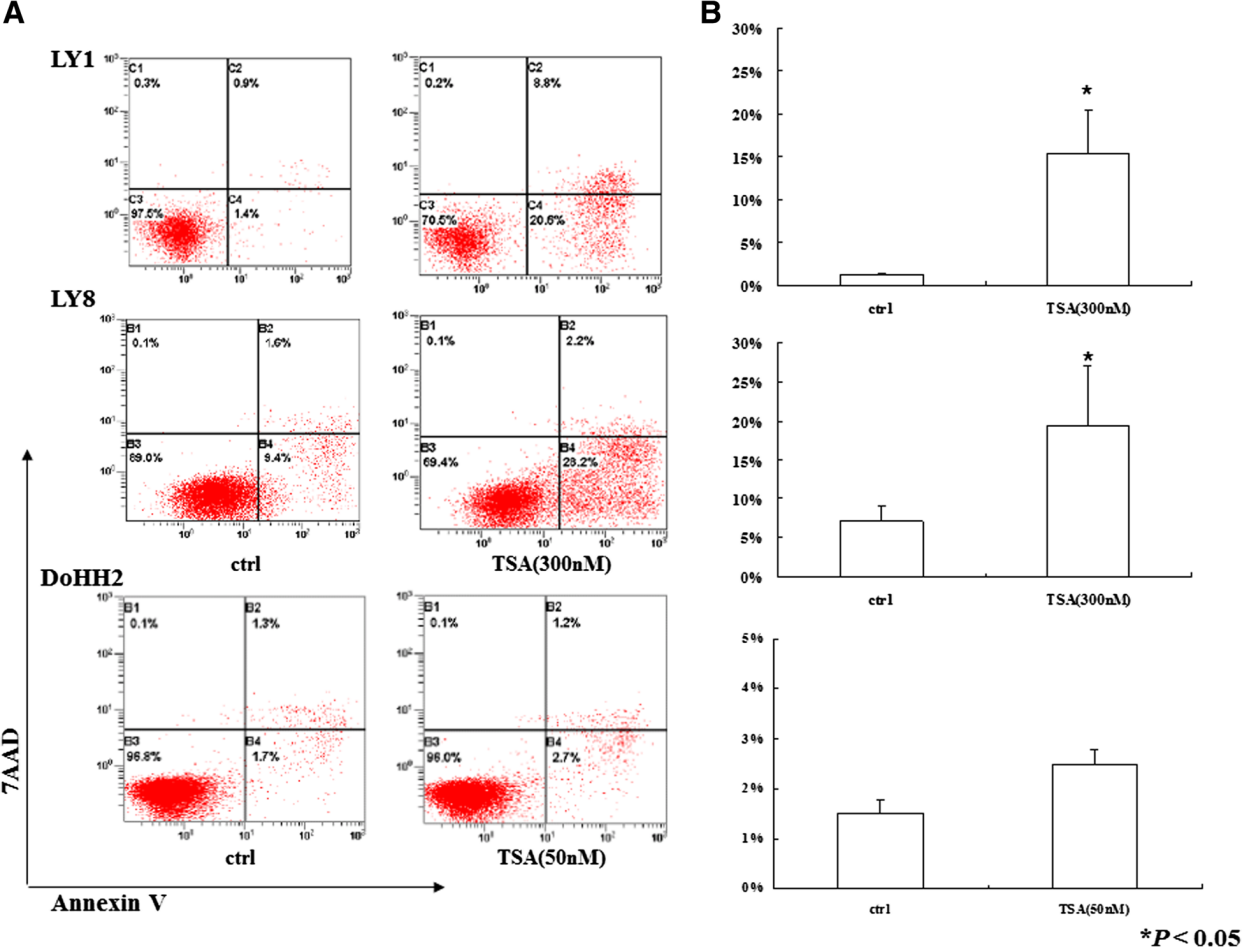
**Effects of TSA on apoptosis in DLBCL cells. **(**A**) Annexin V/PE-7AAD dual staining and flow cytometric analysis of early and late apoptosis of LY1, LY8 and DoHH2 cells treated with TSA (concentrations as indicated) for 24 h. One representative experiment shown for each cell line. The lower right quadrant shows percentage of cells in early apoptosis, the upper right quadrant indicates percentage of cells in late apoptosis, and the lower left quadrant shows percentage of vital cells. (**B**) Percentages of early apoptosis cells are displayed as mean ± SD of three independent experiments performed in triplicate. * *P* < 0.05 compared with vehicle treatment.

The Annexin V/PE-7AAD dual staining assay showed that 24 h treatment with TSA induced apoptosis in both LY1 cells (1.4% vs. 20.6%) and LY8 cells (9.4% vs. 28.2%) (Figure 3A). As shown in Figure 3B, significant apoptosis was induced in LY1 and LY8 cells after 24 h TSA exposure relative to control groups (*P* < 0.05). Furthermore, apoptosis occurred earlier in LY8 cells than in LY1 cells (18 h vs. 24 h, data not shown). However, no significant apoptosis was observed in DoHH2 cells upon TSA treatment (50 nM) (Figure 3B).

### HDAC expression in DLBCL cell lines

**Figure 4:**
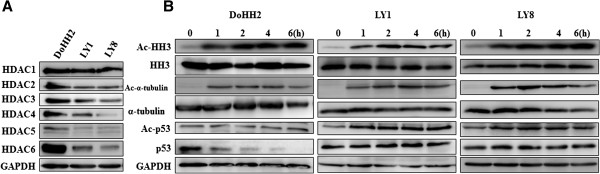
**Expression of HDAC1–6 in DLBCL cells and effects of TSA on its substrates.** (**A**) Expression of HDAC1–6 in three DLBCL cell lines by western blot analysis. (**B**) Expression of substrates of TSA, including histone H3, α-tubulin and p53, and their acetylation patterns at different time points after TSA treatment.

We next determined the expression profile of the main HDAC isoforms in each cell line. Western blot analysis revealed differential expression levels of Class I HDACs (HDAC1, 2 and 3) and Class II HDACs (HDAC4, 5 and 6) in the three DLBCL lines (Figure 4A). All three cell lines strongly expressed HDAC1 and HDAC2. Higher expression levels of HDAC3 and HDAC4 were found in DoHH2 and LY1 cells compared to LY8 cells. HDAC5 was only found in DoHH2 cells and at very high levels. DoHH2 cells also expressed the highest levels of HDAC6, while moderate to weak expression was observed in LY1 and LY8 cells. Together these data showed that the highest expression levels of all six HDAC isoforms were detected in DoHH2 cells, suggesting that the high sensitivity to TSA in DoHH2 cells might be due to the high expression of HDACs.

### TSA-induced acetylation of histone and non-histone proteins in DLBCL cells

To further examine the effects of TSA, we evaluated acetylation of HDAC-related biomarkers, histone H3 and α-tubulin. Histone H3 (HH3) is one of the main substrates of Class I HDAC and α-tubulin is a target of HDAC6. Both acetyl-histone H3 and acetyl-α-tubulin levels were elevated in the three cell lines after 1 h treatment (Figure 4B), suggesting that TSA could inhibit their deacetylation. Though a non-histone protein, p53 is also a substrate of HDAC and its acetylation enhances its stability and extends its half life [8]. Alterations of acetyl-p53 levels were found in LY1 and LY8 cells (Figure 4B). After 1 h incubation with TSA (300 nM), acetyl-p53 levels increased in LY1 and LY8 cells, which express mutant p53 [9]. In contrast, in DoHH2 cells, which express wild-type p53 [10], 50 nM TSA did not cause any apparent changes in acetyl-p53 levels and downregulated p53 expression (Figure 4B).

### Dephosphorylation of pAkt and subsequent negative regulation of its downstream effectors p21, p27 and cyclin D1 after TSA treatment

**Figure 5:**
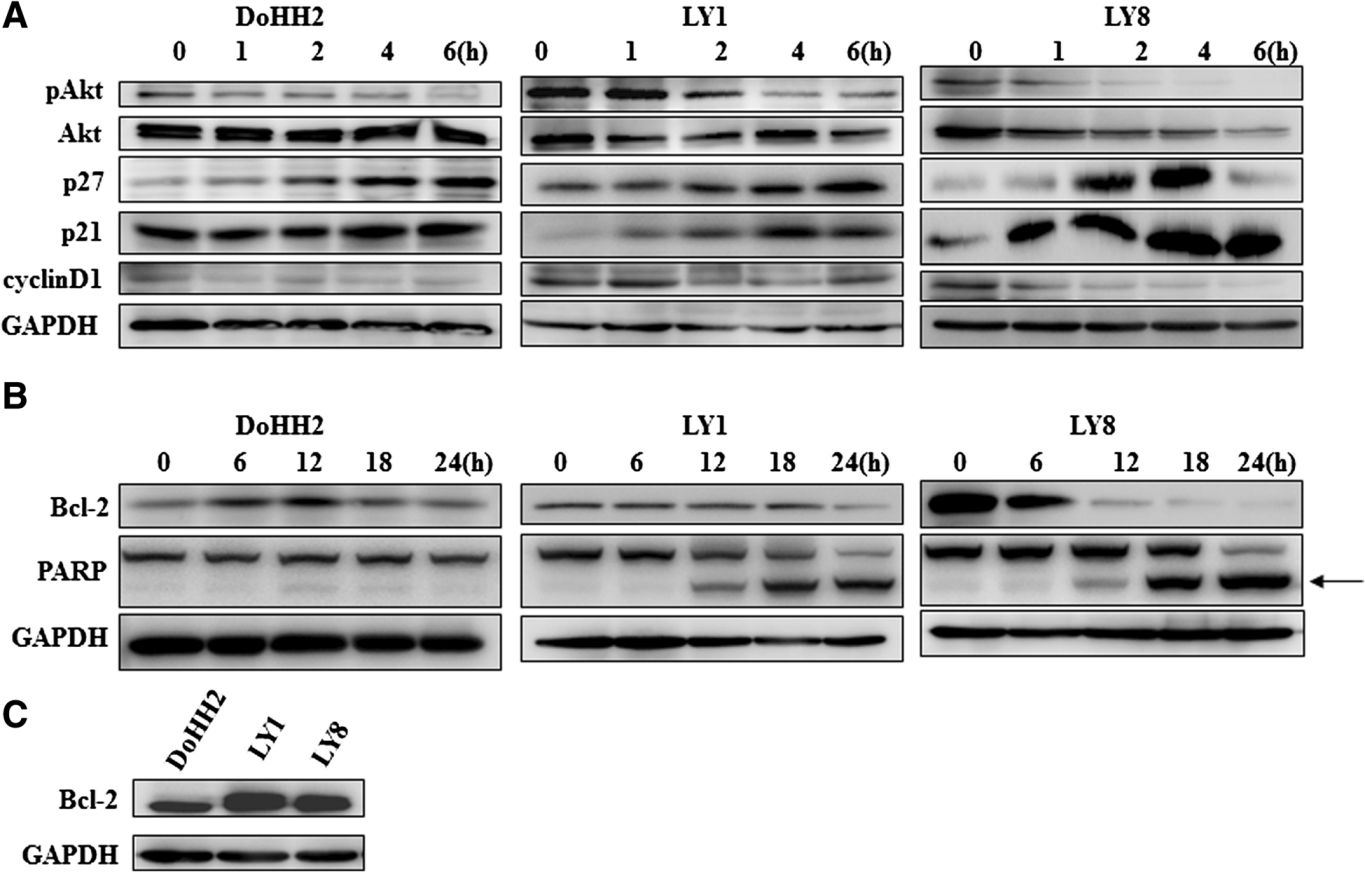
**Effects of TSA on cell cycle arrest and apoptosis proteins in DLBCL cells. **(**A**) Effect of TSA in DoHH2 (50 nM), LY1 and LY8 cells (300 nM) on pAkt and its downstream targets as assessed by western blot. (**B**) Western blot analysis of apoptosis regulators Bcl-2 and PARP in TSA-treated cells. Arrowhead indicates the cleaved product of PARP. (**C**) Expression of Bcl-2 in DLBCL cell lines.

Overexpression of pAkt is commonly observed in DLBCL [11]. After TSA treatment, downregulation of pAkt was consistently detected in all three cells lines (Figure 5A). Both p21 and p27, downstream targets of pAkt, showed variable expression in the three cell lines (Figure 5A). Levels of p27 were continually upregulated and peaked at 6 h in DoHH2 and LY1 cells. In LY8 cells, expression of p27 increased after 2 h and declined after 6 h of TSA exposure. Expression of p21 significantly increased after 1 h incubation with TSA in LY1 and LY8 cells, while DoHH2 cells showed no apparent changes in p21 levels. Cyclin D1, another downstream effector in the Akt pathway, was downregulated in LY1 and LY8 cells, but not in DoHH2 cells.

### Downregulation of Bcl-2 and cleavage of PARP induced by TSA

Bcl-2, an anti-apoptotic protein, was previously reported to be overexpressed in DLBCL [12], which was confirmed in the cell lines we tested (Figure 5C). We next examined the expression level of Bcl-2 before and after TSA treatment. As indicated in Figure 5B, we found downregulated Bcl-2 expression levels in LY1 and LY8 cells after TSA treatment with earlier peak levels in LY8 cells, in which the apoptotic response was detected earlier than in LY1 cells. However, in DoHH2 cells, Bcl-2 was upregulated only for 12 h and then returned to previous levels. PARP is a 116 kDa nuclear poly (ADP-ribose) polymerase, and its cleaved fragment serves as a marker for cells undergoing apoptosis. Cleaved PARP (89 kDa) was found in LY1 and LY8 cells in which apoptosis was detected by Annexin V/PE-7AAD dual staining, while no cleaved fragment was detected in DoHH2 cells, in which apoptosis did not occur (Figure 5B).

## Discussion

Epigenetic regulation of gene expression via acetylation of histone and non-histone proteins is a new and promising therapeutic strategy [13]. Despite research of proposed mechanisms of the anti-proliferative effects of HDAC inhibitors on lymphoid malignancies [14,15], the exact effects and mechanisms in DLBCL remain unclear. Treatment and clinical trials of lymphoma using HDAC inhibitors remains empiric.

To obtain insights into the mechanisms and specificity of HDAC inhibitors toward lymphoma cells, we treated three DLBCL cell lines with a pan-HDAC inhibitor, TSA. TSA, which has a chemical structure similar to Vorinostat (Zolinza), is a hydroxamate-based agent that belongs to the largest group of HDACi. It has been reported to have pleiotropic effects on tumor cells and suppresses cell growth, which contributes to its pan-HDAC inhibitory properties. Although its side effects and toxicity have limited its clinical use [16], TSA is still an ideal tool and representative of the pan-HDAC inhibitors used to analyze the underlying mechanisms of the anti-proliferation effects of these inhibitors in in vitro studies.

TSA was found to exert a potent anticancer activity on human tongue squamous cell carcinoma cells [17]. Another in vitro study in prostate cancer cells showed that TSA led to G_2_/M cell cycle disruption and apoptosis in LNCaP cells [18]. TSA was also reported to inhibit the growth of uveal melanoma cells with a significant reduction of viable cells and increased apoptosis [19]. In our study, we demonstrated the growth inhibitory effects of TSA in three DLBCL cell lines, both in a dose-dependent and time-dependent manner. Cell cycle arrest in G_0_/G_1_ phase was observed in treated DoHH2 and LY1 cells, while a significant G_2_/M phase delay was seen in LY8 cells, in which apoptosis occurred earlier compared to the other two cell lines. Cell cycle arrest and apoptosis may be the basis for the subsequent growth inhibition observed in these cells. The increasing evidence of anti-proliferation effects of hydroxamate-based HDAC inhibitors indicates these to be a category of promising anti-tumor agents.

Aberrant expression of HDACs has been previously detected by immunostaining in various tumors [2]. However, only hematological malignancies appear to be particularly sensitive to HDAC inhibitor therapy [13]. Expression of HDACs in lymphoid malignancies was previously reported [3,20]. Gloghini et al. evaluated the expression of HDAC class 1 and 2 in cell lines and primary tissues from different histotypes of human lymphomas and found the most frequently altered HDAC expression was HDAC6 [3]. High expression of HDAC6 correlated with a favorable outcome in CTCL [20]. In a more recent study, Marquard et al. discovered a correlation between favorable outcome and moderate to strong HDAC6 expression in DLBCL patients [21]. However, the mechanisms underlying HDAC6 effects on patients’ survival remains unknown. In this study, our expression profiling of HDAC1–6 in three lymphoma cell lines found the highest expression level of all six isoforms in DoHH2 cells, which were more sensitive to TSA. Our results suggest that HDAC expression level may correlate with HDAC inhibitor sensitivity. Among all six isoforms, HDAC6 displayed significant variability in all three cell lines. The correlation between high HDAC6 levels in DLBCL cells and sensitivity to TSA should be further investigated with RNAi-mediated knockdown of HDAC6 to examine whether the knockdown reverses the sensitivity. HDAC6 is one of the targets of pan-HDACi. Its high expression in DLBCL suggests HDAC6 might be a potential therapeutic target for the treatment of lymphoid malignancies, since it plays a critical role in the cellular clearance of misfolded proteins via formation of aggresomes and autophagy [22,23]. Tubacin, a selective HDAC6 inhibitor, has been reported to have anti-proliferative effects and induce apoptosis in acute lymphoblastic leukemia (ALL) cells. Treatment with tubacin led to the induction of apoptotic pathways in both pre-B and T cell ALL cells [24] and induced EBV-positive Burkitt lymphoma cell death [25]. The effects of HDAC6-selective inhibitors on DLBCL cells, however, had been previously unclear and the exact function of HDAC6 in DLBCL had remained unknown.

The p53 transcription factor, a non-histone protein, is another substrate of HDACs. In our study, p53 acetylation at Lys382 was higher in LY1 and LY8 cells. Mutation of p53 gene is a common genetic alteration in lymphoma [26]. LY1 and LY8 cells harbor a mutated form of p53, but the mutation did not interfere with the observed enhanced acetylation at Lys382. These cells exhibited stable expression levels of mutant p53, and its acetylation increased in response to TSA. According to the allosteric model, acetylation of p53 causes p53 conformational changes to activate the DNA binding domain and induce enhanced transcriptional activity [27], leading to activation of cell cycle arrest and apoptosis. However, Yan et al. reported that mutant p53 transcription was suppressed by HDACi via HDAC8 in HaCaT cells (human keratinocyte cells) and SW480 cells (human colorectal cells) [28]. These cell lines contain p53 mutants different from LY1 and LY8 cells, with mutations distinct from p53 acetylation sites. Acetylation of wild-type p53 increases its stability [8]. However, no obvious upregulation of acetyl-p53 was observed in DoHH2 cells after TSA treatment, and the level of wild-type p53 protein appeared to be unstable and declined in a time-dependent manner. Alcendor et al. reported a similar phenomenon in their research, showing that p53 acetylation as well as transcriptional activity of p53 was not increased by TSA in cardiac myocytes [29]. Decrease of wild-type p53 protein might be due to the regulation of HDAC inhibitors on p53 transcription. Peltonen et al. discovered that TSA stabilized wild-type p53 in melanoma cell lines, but p53 protein accumulation was overridden by simultaneous downregulation of p53 mRNA, leading to a decrease in p53 protein [30]. The mechanisms of p53 acetylation on both wild-type and mutant proteins in different tumors after various HDACi exposure requires further investigation.

The Akt pathway plays an important role in cell growth, and its activation is common in tumors [31]. Inhibition of overphosphorylated Akt is a promising target therapy in colorectal cancer [32]. We observed pAkt overexpression in all three cell lines and subsequent downregulation after TSA treatment. A similar phenomenon was reported in other studies. Chen et al. demonstrated that HDACi caused Akt dephosphorylation in U87MG glioblastoma and PC-3 prostate cancer cells by disrupting HDAC-protein phosphatase 1 (PP1) complexes [33]. LBH, another HDACi with a chemical structure similar to TSA, mediated Akt dephosphorylation in DLBCL DHL-6 cells through increased binding of PP1 to Akt [34]. We further studied the downstream targets in the Akt pathway. Upregulation of p21 was previously commonly reported, with less data on p27 [14]. Repression of cyclin D1 from HDAC inhibitors was reported in mantle cell lymphoma [35]. In our study, we found more significant alterations of p27 and cyclin D1 than p21 after TSA treatment. Both p21 and p27 were upregulated, and cyclin D1 was downregulated with decreasing expression of pAkt, which may account for the eventual cell cycle delay.

TSA also induces cell apoptosis in LY1 and LY8 cells. Bcl-2, an anti-apoptosis regulator, was found to be downregulated after TSA treatment in LY1 and LY8 cells. In normal germinal centers, Bcl-2 is usually inactivated, rendering centroblasts and centrocytes susceptible to apoptosis. Abnormal retention of Bcl-2 leads to cells that do not die, thereby predisposing cells to malignant transformation. In our study, western blot analysis showed that the repression of Bcl-2 occurred at the translational level in LY1 and LY8 cells after TSA treatment. Its downregulation may be the combined effect of Akt dephosphorylation and p53 acetylation caused by TSA. However, Bcl-2 alteration in DoHH2 cells was quite different with LY1 and LY8 cells. Bcl-2 gene rearrangement was previously reported in DoHH2, LY1 and LY8 cells [36,37]. However, there is no detailed information regarding Bcl-2 amplification in the literature. Our unpublished data showed that all three cell lines do not have apparent Bcl-2 gene amplification. One reason for the differential effects on Bcl-2 may be due to different levels of p53 acetylation. Low p53 acetylation may contribute to DoHH2 cells’ resistance to apoptosis after TSA treatment at IC_50_. The exact mechanisms underlying this process need to be further investigated.

## Conclusion

This investigation addressed the inhibitory effects and underlying mechanisms of TSA, a pan-HDAC inhibitor, in DLBCL cells. TSA suppressed the growth of all three DLBCL cell lines by enhanced G_0_/G_1_ or G_2_/M arrest and possible apoptosis. Expression levels of HDACs varied in the three cell lines, with DoHH2 cells exhibiting the highest expression of all six isoforms of HDAC1–6.The expression levels of HDACs may be associated with TSA sensitivity. Upregulated acetylation of histone H3, α-tubulin and p53 and dephosphorylation of pAkt with alterations of its main downstream effectors (p21, p27, cyclin D1 and Bcl-2 proteins) suggested that inhibition of Akt and activation of the p53 pathway may be the main molecular events involved in the TSA inhibitory effects. Our results have offered evidence supporting the development of HDAC inhibitors to combat DLBCL more efficiently. Studies in more DLBCL cell lines treated with different HDACi are needed to provide more substantial evidence and clarify the roles and mechanisms of HDACi on DLBCL to enhance their clinical applicability.

## Methods

### Cell lines and culture conditions

Three human DLBCL cell lines, LY1, LY8 and DoHH2, were used in this study. LY1 and LY8 cells were kindly provided by Dr B. Hilda Ye (Albert Einstein College of Medicine, USA) and grown in IMDM medium (Gibco, Grand Island, USA) supplemented with 10% FBS (HyClone, Logan, USA). DoHH2 cells were a gift from Prof. Mingzhi Zhang (The First Affiliated Hospital of Zhengzhou University, PRC) and cultured in RPMI1640 (HyClone) containing 10% FBS. Cells were grown and maintained at 37°C in a 5% CO_2_ humidified atmosphere.

### Reagents and treatments

TSA (Sigma-Aldrich, Taufkirchen, Germany) was dissolved in DMSO (Sigma-Aldrich) as a 5 μM stock solution, aliquoted and stored at -20°C. Control cells were treated with DMSO and analyzed in parallel in each experiment.

DoHH2, LY1 and LY8 cells were treated with TSA at concentrations ranging from 5 nM to 1000 nM for 24–72 h. Cell viability was determined by the cell counting kit-8 (CCK-8, Dojindo, Kumamoto, Japan) and fifty percent growth inhibition (IC_50_) of a 48 h TSA exposure in each cell line was obtained from Probit Regression using SPSS16.0. From these results, we selected the following treatment levels for subsequent experiments: 50 nM TSA for DoHH2 cells, and 300 nM TSA for LY1 and LY8 cells.

### Cell proliferation assay

Cell proliferation was assessed using the CCK-8 assay according to the manufacturer’s instructions. Cells (100 μl, 5 × 10^5^ cells/ml) were seeded into a 96-well plate and cultured in RPMI1640 supplemented with 10% FBS containing concentrations of TSA ranging from 0 to 1000 nM. The plate was incubated in a humidified incubator (37°C, 5% CO_2_) for 24–72 h. Four hours before measuring the absorbance, 10 μl of the CCK-8 solution was added into each well. Cell viability was obtained as the percentage of viable cells relative to untreated cells under the absorbance at 450 nm in a microplate reader (Biotek Instrument, Winooski, USA). Two control wells without cells were prepared and average absorbance of the control wells was subtracted from that of the corresponding sample wells. Each experiment was performed in triplicate.

### Cell cycle analysis

Cells incubated with or without TSA were fixed gently in absolute ethanol overnight at -20°C. After resuspension in PBS containing 5 μg/mL propidium iodide (Sigma) and 100 μg/ml RNase A (Takara Biotechnology, Dalian, China), cells were incubated in the dark for 15 min at room temperature and subjected to analysis on a Flow Cytometer Cytomics FC500 (Beckman Coulter, Brea, USA). A total of 3 × 10^4^ events were counted from each sample. Cell cycle distribution was calculated using CXP Software (Beckman Coulter), with the number of gated cells in G_1_, S and G_2_ phase presented as a percentage. Each experiment was performed in triplicate.

### Apoptosis assay

After incubation with or without TSA, cells were harvested at the indicated time. Apoptotic populations were quantified using the dual staining Annexin V/PE-7AAD apoptosis detection kit (BD Biosciences, San Diego, USA) according to the manufacturer’s instructions before flow cytometric analysis. At least 1.5 × 10^4^ events were counted. The percentage of apoptotic cells in each quadrant was calculated using CXP Software. Each experiment was performed in triplicate.

### Western blot analysis

Cells were harvested and lysed, and total protein concentrations of cell lysates were determined by the BCA Protein Assay Kit (Pierce Biotechnology, Rockford, USA). Protein samples (30 μg) were separated by 12% SDS-PAGE and transferred onto a polyvinylidene fluoride membrane (Millipore, Billerica, USA). The membrane was blocked in Tris-buffered saline containing 5% bovine serum albumin and 0.1% Tween at room temperature for 3 h, incubated with diluted primary antibody overnight at 4°C with gentle shaking, and then incubated with secondary antibody for 1 h at room temperature. The following primary antibodies were used for analysis: Ac-Histone H3 (Lys9, C5B11), Histone H3 (D1H2), Ac-α-tubulin (Lys40, D20G3), α-tubulin (11H10) Ac-p53 (Lys382), pAkt (Ser473, 193H12), Akt (40D4), Bcl-2 (50E3), p21 (12D1), p27 (SX53G8.5), cyclin D1 (DCS6), PARP (46D11), HDAC1 (10E2), HDAC2 (3F3), HDAC3 (7G6C), HDAC4 (D15C3), HDAC5 and HDAC6 (D2E5), all from Cell Signaling Technology (Danvers, USA). Anti-p53 antibody (C-11) that recognizes full-length p53 was purchased from Santa Cruz Biotechnology (Santa Cruz, USA). The anti-rabbit IgG and anti-mouse IgG secondary antibodies were purchased from Cell Signaling Technology. Signals were developed with enhanced chemiluminescence substrates (Pierce Biotechnology) according to the manufacturer’s protocols and visualized by Image Quant LAS 4000 (GE Healthcare Biosciences AB, Uppsala, Sweden). GAPDH (Proteintech Group, Chicago, USA) served as a loading control.

### Statistical analysis

All cell culture experiments were repeated three times with similar results. Data were presented as mean ± SD. Statistical comparisons were made using an unpaired 2-tailed Student’s t-test between different groups. SPSS16.0 software (Chicago, USA) was used to perform statistical analysis. Statistical significance was set at *P* value of < 0.05.

## Competing interests

The authors declare that they have no competing interests.

## Authors’ contributions

Y Cai performed most of the experiments. W Cui and W Chen coordinated the study. P Wei and Y Chi evaluated all immunoblots. P Zhang performed the cell cycle and apoptosis assays. R Bi provided important help in statistical analysis. X Zhou designed and coordinated the study. Y Cai and X Zhou wrote the manuscript. All authors read and approved the final manuscript.
